# Successful healing of chronic venous ulcer using zinc oxide nanoparticles and compression therapy in a 75-year-old Syrian female: a case report

**DOI:** 10.1097/MS9.0000000000002881

**Published:** 2025-01-09

**Authors:** Hiba Shreiki, George Bashour, Houssam Kinjo

**Affiliations:** a Faculty of Pharmacy, Tishreen University, Latakia, Syria; b Faculty of Medicine, Tishreen University, Latakia, Syria; c Department of Vascular Surgery, Tishreen University Hospital, Latakia, Syria

**Keywords:** case report, chronic wounds, compression therapy, nanoparticle drugs, venous ulcer, zinc oxide

## Abstract

**Introduction:**

Chronic venous disease is a commonly underdiagnosed condition that gradually diminishes a patient’s quality of life and imposes a growing burden on healthcare resources. Venous leg ulcers (VLUs) arise as a complication of chronic venous insufficiency and represent the most prevalent type of slow-healing wound in the lower extremities.

**Case presentation:**

In this case report, we present the successful treatment of a 75-year-old woman with a chronic venous ulcer caused by chronic deep venous insufficiency. The patient had been struggling with an unresponsive venous leg ulcer for 7 years with no improvement from previous treatments. A three-layer Unna boot treatment, including (ZnO NPs) and a two-component compression system was applied, and significant ulcer reduction was observed.

**Discussion:**

ZnO NPs impregnated bandages, like those found in an Unna boot, offer compression by wrapping gauze dressing impregnated with ZnO NPs around the patient’s leg forming a semi-solid mold around the extremity. A more efficient treatment approach could lead to fewer clinic visits, thereby reducing healthcare expenses by enhancing the method of managing venous ulcers through a combination of compression and simple, cost-effective surface dressing materials. It’s worth noting that ZnO NPs have demonstrated significant benefits for wound healing.

**Conclusion:**

Utilizing ZnO NPs alongside compression therapy has proven highly effective, accelerating healing and offering a cost-effective solution. Further research is needed to study the safety of ZnO NPs.

## Introduction

Chronic ulcers are those that are unable to heal through the usual process due to a Continuous inflammatory phase. Among the many types of chronic wounds, one of the most prevalent types is chronic venous leg ulcer (VLU), which affects approximately 2%–3% of the population. The commonality of VLUs leads to significant effects on public health programs, resulting in increased expenses, prolonged treatments, and disabilities. VLUs have negative effects on the quality of life and productivity of individuals, causing pain, limited mobility, social isolation, and depression[1].

VLUs are known for their slow healing process, which leads patients to suffer from open wounds for several months. Over 50% of ulcers that persist for 6 weeks remain unrecovered even after 1 year[2].

Compression therapy is considered the primary treatment for VLUs. It promotes healing by restoring valve function, reducing venous hypertension and reflux, and improving lymphatic drainage from the affected limb. This leads to the resolution of pain and edema associated with VLUs[3].

The occurrence of chronic wounds including (venous, diabetic foot, or pressure ulcers) is nearing epidemic levels, which highlights the need to find more efficient solutions[4].

Nanotechnology, using nanomaterials, has introduced a new concept in wound healing, facilitating faster healing practices and exhibiting unique antibacterial properties[5]. The inability of several novel approaches to accomplish wound closure and fluid loss control, along with the need for materials that possess attributes like durability, elasticity, and histocompatibility, has resulted in the development of various nanotechnological advancements[4].

Zinc oxide is a crucial metal oxide utilized in a range of biological applications, ZnO nanoparticles (ZnO NPs) have diverse biomedical applications through the interaction between the nanoparticles, which affect their biomedical applications[6]

In this case report, we added a layer of ZnO NPs paste bandage to the multi-layer compression therapy to monitor the healing progression in an unresponsive VLU in a 75-year-old Syrian female.

This case report has been reported in line with the SCARE criteria[7].

## Case representation

**Figure 1.**
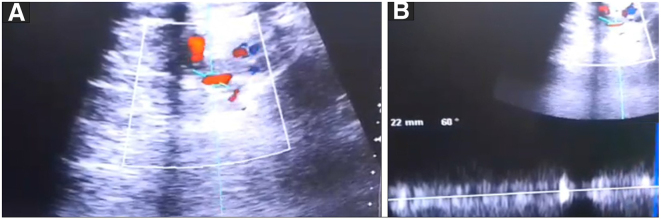
Doppler ultrasound which shows the deep venous insufficiency wave at the level of the left femoral vein.

A 75-year-old woman presented to the vascular clinic with a chronic venous ulcer induced by deep venous insufficiency (DVI) on the medial side of her left Leg, which has not responded to treatment for 7 years. The diagnosis was made according to the CEAP classification as follows: C6s Ep Ad Pr/femoral and popliteal insufficiency. The patient complained about severe pain and inability to walk properly. The patient’s history included hypertension and previous treatments of the ulcer (Topical Ozon creams, Fusidic acid creams, and honey bandages) without any improvements. She was previously put on Tramadol to manage her pain and rivaroxaban (10 mg) as a preventive does for the DVI. Additionally, the patient underwent saphenous vein resection before the venous insufficiency diagnosis. The ulcer was reassessed with Doppler ultrasound during clinical examination, and all previous treatments were discontinued (Fig. 1A and B).

**Figure 2.**
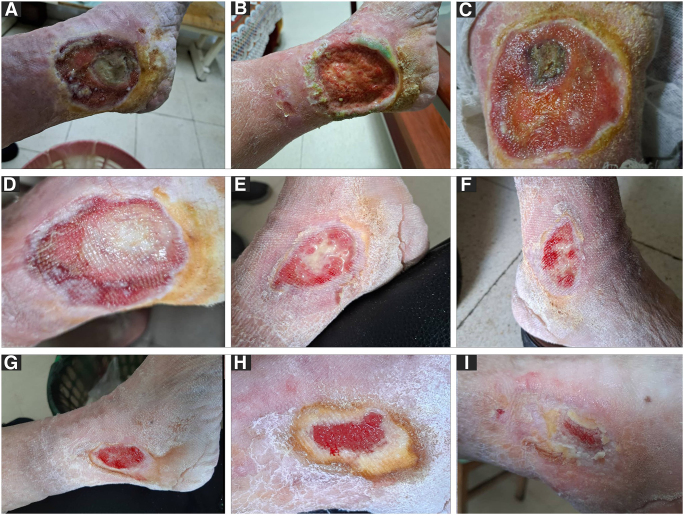
(A) The ulcer at presentation. (B) After the initial surgical debridement, where the formation of the granulation tissue started. (C) Three weeks after treatment, the second surgical debridement removed the necrotic center area shown in this image. (D–F) The ulcer progression after 4, 5, and 6 weeks of treatment. (G–I) The ulcer progression during the last month of treatment in order by intervals of 10 days with the last image (I) showing over 95% healing at 3 months.

The wound was cleansed with a sterile saline solution and wide surgical debridement was performed (Fig. 2A and B).

Then, a three-layer Unna boot was applied. The ZnO NPs paste bandage layer was placed directly on the ulcer and to manage the exudate, an absorbent pad was placed over the zinc layer before applying the compressive layer. The cohesive compression bandage served as the outer layer, adhered to itself or the inner bandage but not to the skin and provided pressure up to 40 mmHg. The bandage was applied from the toes to 3 inches below the knee. Tramadol was replaced with pregabalin (75 mg po bid) for 2 weeks and oral antibiotics were given for 1 week. Daflon (500 mg) was also prescribed. The bandage was replaced every week for the first month, every ten days in the second month and every 15 days in the third month which accumulated to nine bandages in total. There was no mobility limitation during the period of the treatment. During the replacement of the third bandage, minor surgical debridement was performed (Fig. 2C). After 9 months of follow-up, no recurrence has occurred and the ulcer healed well.

## Discussion

On a global scale, 1%–2% of adults have ulcers in their lower limbs, and the proportion increases to 3% for 70%–90% of patients aged 65 years and older[8]. Research has shown that compression therapy improves healing rates significantly. Additionally, multi-component systems were more effective than single-component systems[8].

The Unna boot is considered to be the primary intervention for venous ulcers,[2] with a treatment rate between 40% and 60% in 3 months, and up to 70% from 6 months to 1 year[9]. It contains a zinc oxide paste bandage, which has been proven effective in the healing process of ulcers[2].

Zinc paste bandage is considered necessary for the treatment of specific types of venous illnesses including thromboembolic diseases, and chronic venous diseases[10]. One of the important types of zinc paste bandage is the type that includes nanoparticles[10].

Metallic nanoparticles have been recognized for clinical usage in the field of wound healing, due to their cost-effectiveness, substantial surface-to-volume ratio, stability, and non-toxic nature[5].

The use of ZnO NPs allows a greater bioavailability of zinc in the wound due to the release of Zn^+^[2] ions, achieving a biocidal effect on bacteria (gram-positive and gram-negative), fungi and algae, favoring the wound healing process[11]. The biomedical applications of ZnO are a result of electron-hole pairs formation, which generate Reactive oxygen species (ROS) and demonstrate antimicrobial properties.it also aids in the re-epithelialization process of wound healing[6].

Another method for wound healing is split-thickness skin grafting (SSG), which includes removing the epidermis and portion of the dermis, leaving the reticular dermis in the donor site, allowing skin healing by secondary intention[12]. Although its important role in wound healing, the donor site transforms into a second wound, often painful and needs more time to heal, with the possibility of infection and scarring.[12]

In this case report, a unique approach involving the application of a three-layer Unna boot with zinc oxide nanoparticles paste bandage layer was used and followed by the application of a compressive bandage.

The concentration of active ZnO NPs was 70%, and the two-component compression bandage consisted of a single-sided cohesive coated elastic bandage that served as a padding layer. It distributed the pressure of the outer layer evenly along the leg to ensure the required pressure.

The combination of ZnO NPs and compression therapy led to a significant reduction in the ulcer within 6 weeks and a near-complete healing at 3 months. This successful outcome highlights the potential of ZnO NPs for wound healing and ulcer treatment.

Utilizing has various biomedical applications, including the usage for skin and leg ulcers, resulting in the acceleration of the healing process when used in the treatment of ulcers[10]. Along with compression therapy, it presents a cost-effective solution for the patient. It reduces clinical visits, thereby lowering public health-related costs and improving the patient’s quality of life.

It is important to note that as the concentration of nano-zinc particles increases, so does the potential for greater toxicity on surrounding tissues. Therefore, it is essential to carefully consider the suitable concentration of nano-zinc particles to minimize any adverse effects on tissue health. Additionally, research has revealed that the size and surface properties of the particles can also have an impact on their toxicity[13].

## Conclusion

Venous leg ulcers impose a significant burden on healthcare systems due to their expenses and prolonged treatment. Therefore, new and effective treatment methods are required to manage them. This report presents the successful treatment of a 7-year-old unresponsive venous ulcer using ZnO NPs paste bandages and two-layer compression bandages. The utilization of this material in the bandages expedited the healing process in conjunction with the compression bandages. Further research with larger sample size is needed to study the safety of ZnO NPs and their effectiveness in treating ulcers.

## Data Availability

The manuscript is case report or case series. There is no research data.

## References

[1] Bello-López JM López-Ornelas A Vilchis-Rangel RE. In vitro bactericidal activity of a carbohydrate polymer with zinc oxide for the treatment of chronic wounds. J Med Microbiol 2020; 69:874–80. 32459619 10.1099/jmm.0.001204

[2] Gao AL Cole JG Woolsey ZT. Unna boot central gauze technique for chronic venous leg ulcers. Dermatol Online J 2017; 23:2024. 28329467

[3] Garavello A Franzvea P Tozzi M. Deep vein insufficiency and the results of four-layer compression bandages in the treatment of venous ulcers: a retrospective study. J Wound Manag 2021; 22:15–19.

[4] Hamdan S Pastar I Drakulich S. Nanotechnology-driven therapeutic interventions in wound healing: potential uses and applications. ACS Cent Sci 2017; 3:163–75. 28386594 10.1021/acscentsci.6b00371 PMC5364456

[5] Mendes C Thirupathi A Corrêa ME. The use of metallic nanoparticles in wound healing: new perspectives. Int J Mol Sci 2022; 23:15376. 36499707 10.3390/ijms232315376 PMC9740811

[6] Kaushik M Niranjan R Thangam R. Investigations on the antimicrobial activity and wound healing potential of ZnO nanoparticles. Appl Surf Sci 2019; 479:1169–77.

[7] Sohrabi C Mathew G Maria N. The SCARE 2023 guideline: updating consensus Surgical CAse REport (SCARE) guidelines. Int J Surg 2023; 109:1136–40. 37013953 10.1097/JS9.0000000000000373 PMC10389401

[8] O’Meara S Cullum N Nelson EA. Compression for venous leg ulcers. Cochrane Database Syst Rev. 2012:11. Accessed June 28, 2024. https://www.cochranelibrary.com/cdsr/doi/10.1002/14651858.CD000265.pub3/abstract 10.1002/14651858.CD000265.pub3 PMC7068175 23152202

[9] Cardoso LV de GJMP de Fg GM. Compression therapy: Unna boot applied to venous injuries: an integrative review of the literature. Rev Esc Enferm USP 2018;52:e03394. 30517291 10.1590/S1980-220X2017047503394

[10] Alwis R Alwis R Al-Raeei M. Nanoparticles zinc paste bandages for the treatment of Syrian woman diabetic patient with ulcers in the foot: case images. Clin Case Rep 2023; 11:e7445. 37255618 10.1002/ccr3.7445 PMC10225613

[11] Loera-Valencia R Neira RE Urbina BP. Evaluation of the therapeutic efficacy of dressings with ZnO nanoparticles in the treatment of diabetic foot ulcers. Biomed Pharmacother 2022; 155:113708. 36162373 10.1016/j.biopha.2022.113708

[12] Kanapathy M Hachach-Haram N Bystrzonowski N. Epidermal grafting versus split-thickness skin grafting for wound healing (EPIGRAAFT): study protocol for a randomised controlled trial. Trials 2016; 17:245. 27185033 10.1186/s13063-016-1352-y PMC4869340

[13] Successful treatment of electric burns in young patients. Google Scholar. Accessed July 1, 2024. https://scholar.google.com/scholar?hl=en&as_sdt=0%2C5&q=Successful+treatment+of+electric+burns+in+young+patients+with+nano-zinc+dressings%3A+A+case+report&btnG=

